# An Evidence-Based Approach to the Management of Functional Dyspepsia Associated With Helicobacter pylori Infection

**DOI:** 10.14740/gr598e

**Published:** 2014-03-14

**Authors:** Shou-Wu Lee, Yen-Chun Peng, Chun-Fang Tung, Teng-Yu Lee, Chi-Sen Chang, Hong-Zen Yeh

**Affiliations:** a Division of Gastroenterology and Hepatology, Department of Internal Medicine, Taichung Veterans General Hospital, Taichung, Taiwan; b Department of Medicine, Chung Shan Medical University, Taichung, Taiwan; c Department of Internal Medicine, National Yang-Ming University School of Medicine, Taipei, Taiwan

**Keywords:** Dyspepsia, *Helicobacter pylori*, Functional dyspepsia

## Abstract

**Background:**

Functional dyspepsia is defined as at least a 3-month history of dyspepsia without structural explanation for the symptoms, and it accounts for most cases of dyspepsia. We designed this study to investigate functional dyspepsia among a Chinese population in Taiwan.

**Methods:**

Data from the medical records of 1,143 adult patients who underwent transoral upper endoscopy for symptoms of dyspepsia in our hospital were retrospectively analyzed between January 2008 and December 2008. Exclusion criteria were structural gastrointestinal and hepatobiliary abnormalities, prior gastric surgery or history of chronic medication use.

**Results:**

Patients were mainly in the third and fourth decades of life. More female patients were noted than male patients (ratio 2:1). The rate of *Helicobacter pylori* infection was 18.5%. The rate of response to therapeutic agents, including proton pump inhibitors, H2-receptor antagonists and prokinetic agents, ranged from 69% to 77% among all cases. Patients who underwent *H. pylori* eradication therapy (88.8%) had a significantly higher rate of symptom improvement than those without *H. pylori* infection (77.5%).

**Conclusion:**

Cases with functional dyspepsia have the characteristics of middle age, female predominance, a relatively lower *H. pylori* infection rate and a positive response to *H. pylori* eradication therapy.

## Introduction

“Dyspepsia” refers to a collection of upper gastrointestinal symptoms that is believed to be common world-wide [1], occurring in approximately 25% of the population each year [2-5]. The causes of dyspepsia are known to be either organic (structural or physiological) or functional (non-organic or non-ulcer). Functional dyspepsia is defined by Rome III criteria as at least a 3-month history of epigastric pain, burning, early satiation or postprandial fullness in which there is no obvious structural explanation for the symptoms [6]. Several previous literature reviews have documented the rate of the types of dyspepsia, and functional dyspepsia accounted for the most cases (up to 60%). Besides, between 20% and 60% of patients with documented functional dyspepsia have *Helicobacter pylori* infection [7], and *H. pylori* eradication therapy is not always effective in cases of functional dyspepsia [8].

However, to the best of our knowledge, no study of functional dyspepsia and its relationship with *H. pylori* infection has been conducted among a Chinese population in Taiwan. The aim of this study was to provide formal evidence of empirical treatment and investigation to help primary care physicians combat dyspepsia in Chinese patients with functional dyspepsia.

## Patients and Methods

Data from the medical records of 1,143 consecutive adult patients older than 20 years who underwent open-access transoral upper endoscopy for symptoms of dyspepsia in our hospital, a 1,155-bed academic urban tertiary-care center, were retrospectively analyzed in 1-year duration, between January 2008 and December 2008. Functional dyspepsia was defined as pain and discomfort centered in the upper abdomen without gastrointestinal structural lesions. Exclusion criteria were as follows: 1) structural abnormalities found by upper endoscopy, including reflux esophagitis, gastritis, peptic ulcers or gastrointestinal malignancy, 2) chronic hepatitis, chronic pancreatitis or gallstones diagnosed by blood examination or image findings, 3) cirrhosis with varices or portal hypertensive gastropathy, 4) prior gastric surgery, 5) use of medications, such as proton pump inhibitors (PPI), H2-receptor antagonists (H2RB), aspirin or other non-steroidal anti-inflammatory drugs in the 3 months prior to the enrollment. Written informed consent for upper endoscopy was obtained from all patients before the procedure.

The characteristics of each patient, including age and gender, were recorded, and all findings of upper endoscopy were confirmed by experienced gastroenterologists to avoid individual diagnostic errors. *H. pylori* status was determined from antral biopsy used in the rapid urease test (CLO test, Delta West, Bentley, Australia), and testing was done at the discretion of the primary gastroenterologists. The patients with *H. pylori* infection underwent standard eradication therapy, including oral PPI 20 mg twice a day, amoxillin 1 g twice a day and klaricid 500 mg twice for 1 week. All patients enrolled in the study received standard-dose PPI (omeprazole 20 mg, lansoprazole 30 mg and pantoprazole 40 mg once per day), H2RB (ranitidine 150 mg and cimetidine 400 mg twice a day) or prokinetic agents, mainly metoclopramide alone, at our outpatient clinic. The efficacy of medications was evaluated during the 1 - 3 month period following endoscopy.

Statistical comparisons were made based on age, therapeutic medications and efficacy of therapy, or between genders, using Pearson’s chi-square test. A P value below 0.05 was considered statistically significant.

## Results

Data collected from the medical records of 1,143 consecutive patients with functional dyspepsia during the 1-year study period are displayed in Table 1. Patients in the third and fourth decades of life accounted for 46.8% of all cases. More female patients were noted than male patients, with a female-to-male ratio of 2:1 in all study cases or patients in each subgroup, except in the patients older than 80 years, where males were predominant.

**Table 1:** Patients’ Data

	Age (years)							Total	P value
	20-29	30-39	40-49	50-59	60-69	70-79	> 80		
Case numbers (prevalence)	189 (16.5%)	186 (16.3%)	281 (24.6%)	254 (22.2%)	137 (12%)	74 (6.5%)	22 (1.9%)	1,143 (100%)	
Gender									
Male	84 (44.4%)	75 (40.3%)	105 (37.4%)	88 (34.6%)	53 (38.7%)	33 (44.6%)	17 (77.3%)	455 (39.8%)	0.04^b^
Female	105 (55.6%)	111 (59.7%)	176 (62.6%)	166 (65.4%)	84 (61.3%)	41 (55.4%)	5 (22.7%)	688 (60..2%)	
H.P.									
Positive	9 ( 7.6%)	23 (20.4%)	44 (24.2%)	35 (19.4%)	19 (18.8%)	7 (15.2%)	2 (15.4%)	139 (18.5%)	0.03^b^
Negative	109 (92.4%)	90 (79.6%)	138 (75.8%)	145 (80.6%)	82 (81.2%)	39 (84.8%)	11 (84.6%)	614 (81.5%)	
Not done	71	73	99	74	36	28	9	390	
Medications									
PPI	92 (48.7%)	91 (49%)	136(48.4%)	127 (50.0%)	70 (51.1%)	40 (54.1%)	6 (27.3%)	562 (49.2%)	0.028^b^
H2RB	51 (27.0%)	54 (29%)	60 (21.4%)	64 (25.2%)	39 (28.5%)	21 (28.4%)	13 (59.1%)	302 (26.4%)	
Prokinetic agents	46 (24.3%)	41 (22%)	85 (30.2%)	63 (24.8%)	28 (20.4%)	13 (17.5%)	3 (73.6%)	279 (24.4%)	
Clinic following									
Improved	47 (70.1%)	58 (75.3%)	109 (78.4%)	108 (78.3%)	65 (74.7%)	37 (78.7%)	11 (68.8%)	435 (76.2%)	0.828^b^
Worsen	20 (29.9%)	19 (24.7%)	30 (21.6%)	30 (21.7%)	22 (25.3%)	10 (21.3%)	5 (31.3%)	136 (23.8%)	
Dropout	122	109	142	116	50	27	6	572	

All P values were analyzed with Pearson chi-square test. H.P.: Helicobacter pylori; H2RB: H2-receptor antagonists; PPI: proton pump inhibit.

The overall rate of *H. pylori* infection was only 18.5% in this study. The youngest patients, those between 20 and 29 years old, had the lowest infection rate (7.6%), whereas the middle-aged cases, those between 40 and 49 years old, had the highest infection rate (24.2%). Almost one-half of the patients received PPI as therapeutic medication, one-fourth received H2RB and the other one-fourth of patients were given medications with prokinetic agents. The rate of response to medication was as high as 76.2% in the patients receiving regular clinical follow-up, among whom the youngest and oldest cases accounted for the lowest rate of symptoms improvement (70.1% and 68.8%, respectively). However, the number of patients lost to follow-up was 572, which was more than one-half of all cases in our study, and the reason why these patients lost clinical follow-up might be mostly due to symptom subsided.

The characteristics of patients who had regular clinical follow-up are summarized in Table 2. There was no significant difference between genders in the rate of response to medication. All 571 patients who received clinical follow-up had similar rates of response whether they took PPI (77.2%), H2RB (77.4%) or prokinetic agents (69.4%). In the 80 patients with *H. pylori* infection, the rate of response to medication was highest in cases receiving prokinetic agents (100%), followed by those receiving H2RB (94.7%) and those who were given PPI (84.9%), but these differences were not significant. In the 329 patients without *H. pylori* infection, the rates of response were: H2RB 80%, PPI 76.8% and prokinetic agents 75.6%.

**Table 2:** Patients’ Clinical Characteristics on Follow-Up

	**Clinic following**		**Total**	**P value**
	**Improved**	**Worsen**		
Gender				
Male	176 (75.5%)	57 (24.5%)	233	0.828
Female	159 (76.6%)	79 (23.4%)	238	
All except dropout				
PPI	251 (77.2%)	74 (22.8%)	325	0.282
H2RB	125 (77.6%)	36 (22.4%)	161	
Prokinetic agents	59 (69.4%)	26 (30.6%)	85	
Total	435 (76.2%)	136 (23.8%)	571	
Cases with H.P. infection				
PPI^a^	45 (84.9%)	8 (15.1%)	53	0.289
H2RB^b^	18 (94.7%)	1 (5.3%)	19	
Prokinetic agents^c^	8 (100.0%)	0	8	
Total^d^	71 (88.8%)	9 (11.2%)	80	
Cases without H.P. infection				
PPI^a^	149 (76.8%)	45 (23.2%)	194	0.789
H2RB^b^	72 (80.0%)	18 (20.0%)	90	
Prokinetic agents^c^	34 (75.6%)	11 (24.4%)	45	
Total^d^	255 (77.5%)	74 (22.5%)	329	

P value of a: 0.203; b: 0.124; c: 0.116; d: 0.025. All P values were analyzed with Pearson chi-square test. H.P.: *Helicobacter pylori*; H2RB: H2-receptor antagonists; PPI: proton pump inhibit.

The rate of response to therapeutic medication was higher in patients who underwent *H. pylori* eradication therapy than in those without *H. pylori* infection, whether they received PPI (84.9% vs. 76.8%), H2RB (94.7% vs. 80%) or prokinetic agents (100% vs. 75.6%). Patients with *H. pylori* infection (88.8%) had a higher responsive rate than those without *H. pylori* infection (77.5%), and the difference was statistically significant (P = 0.025, < 0.05). No allergic reactions were recorded in patients who received the *H. pylori* eradication therapy.

## Discussion

“Dyspepsia” refers to a collection of upper gastrointestinal symptoms. Most cases of dyspepsia in previous reports were of the functional subtype (50%-70%) [1]. Rome III criteria defined functional dyspepsia as at least a 3-month history of epigastric pain, burning, early satiation or postprandial fullness in which there is no obvious structural explanation for the symptoms, and these symptoms must be onset at least 6 months before diagnosis [6]. The precise pathophysiology of this condition remains unclear, but it is thought to result from a combination of visceral hypersensitivity, gastric motor dysfunction and psychological factors [9].

The prevalence of functional dyspepsia appeared to peak in Chinese subjects aged 41-50 years [10], and in Japanese adults aged 50-59 years [11]. In our patients with functional dyspepsia, the peak prevalence occurred in those who were 40-60 years old, which was compatible with previous reports. Some population studies have noted a consistent female preponderance with functional dyspepsia [12, 13]. In a previous study of 2,018 health check attendees, female gender was found to be the only independent risk factor for functional dyspepsia [14]. Similarly, our patients showed a parallel pattern, with female predominance in patients with functional dyspepsia. The only exception, that of male predominance in patients older than 80 years, may be due to the characteristics of our hospital, which is a veterans’ hospital, and thus may be less representative because of small case numbers.

According to a previous study, between 30% and 65% of patients diagnosed with functional dyspepsia had *H. pylori*-induced gastritis [15]. However, the association between *H. pylori* and functional dyspepsia remains unclear, and there is no association between *H. pylori* and any specific symptom profile in functional dyspepsia [16]. Our patients had a lower ratio of *H. pylori* infection and this may be because of the relatively high sanitation level in our country.

Once a diagnosis of functional dyspepsia is confirmed by a negative endoscopy and other image findings, an empirical therapeutic trial is commonly prescribed. However, the benefits of all therapeutic modalities for this condition have been questioned [17]. A Cochrane review of eight trials of PPI or H2RB with 1,125 patients showed a relative risk reduction of 30% [18]. An economic model suggested that PPI therapy was cost-effective for functional dyspepsia [19]. Although a study of a US population showed patients benefited significantly from PPI usage [20], a randomized trial of PPI versus a placebo in a study of 453 patients from Hong Kong had an unfavorable outcome [21]. Prokinetics, such as metoclopramide, erythromycin or tegaserod, have limited or poorly established efficacy in patients with functional dyspepsia, because use of these agents to improve gastric emptying does not correlate well with symptom improvement [17]. Our patients reported that PPI, H2RB and prokinetic agents provided similar beneficial effects for the relief of the symptoms of functional dyspepsia, but a placebo group was not included in our study design for comparison.

Previous studies documented that *H. pylori* status is unlikely to affect the therapeutic outcome of acid suppression therapy in cases of functional dyspepsia, so there is no significant difference between the therapeutic outcome in *H. pylori*-positive versus *H. pylori-*negative patients [20]. One recent meta-analysis suggested that a small but significant therapeutic gain can be achieved with *H*. *pylori* eradication [8]. Our study showed a significant improvement of symptoms in the patients who underwent *H. pylori* eradication compared with those without *H. pylori* infection. The results of our study not only provide solid evidence of the efficacy of *H*. *pylori* eradication therapy in infected patients with functional dyspepsia, but also imply that *H. pylori* eradication therapy may be beneficial for patients with uninvestigated dyspepsia who have *H. pylori* infection. Moreover, *H. pylori* eradication in those with functional dyspepsia also helps prevent ulcer disease [16].

There were some limitations in our study. Firstly, our study, a form of referral-based endoscopy, as opposed to a population-based study, may be less representative of the general population due to selection bias. Secondly, although disease of chronic hepatitis, pancreatitis or gallstones are excluded by blood examination and image findings, there are still other causes of dyspepsia which we did not take into account, such as medications, metabolic disturbances and intestinal angina. Including these causes would result in overestimation of the proportion of our patients with functional dyspepsia. Lastly, other major limitations of this study included the retrospective study design, the lack of randomization and the lack of a group of patients receiving placebo medications. Although the duration of this study was designed to be as long as 1 year, it is possible that there were unmeasured differences among subgroups.

### Conclusion

Functional dyspepsia is defined as at least a 3-month history of dyspepsia in which there is no obvious structural explanation for the symptoms. Chinese patients in Taiwan with functional dyspepsia have the characteristics of middle age, female predominance, relatively lower *H. pylori* infection rate and a positive response to *H. pylori* eradication therapy.
